# Does AMH Reflect Follicle Number Similarly in Women with and without PCOS?

**DOI:** 10.1371/journal.pone.0146739

**Published:** 2016-01-22

**Authors:** Sverre C. Christiansen, Tina B. Eilertsen, Eszter Vanky, Sven M. Carlsen

**Affiliations:** 1 Department of Endocrinology, St Olavs Hospital, Trondheim University Hospital, Trondheim, Norway; 2 Department of Cancer Research and Molecular Medicine, Norwegian University of Science and Technology, Trondheim, Norway; 3 Department of Obstetrics and Gynaecology, Hospital of Namsos, Nord-Trøndelag Hospital Trust, Namsos, Norway; 4 Department of Laboratory Medicine, Children’s and Women’s Health, Norwegian University of Science and Technology, Trondheim, Norway; 5 Department of Obstetrics and Gynecology, St Olav’s Hospital, Trondheim University Hospital, Trondheim, Norway; Zhejiang University, CHINA

## Abstract

**Context:**

Increased Anti-Mullerian Hormone in polycystic ovary syndrome, may be due to overactive follicles rather than reflect antral follicle count.

**Objective:**

Does Anti-Mullerian Hormone reflect antral follicle count similarly in women with or without polycystic ovary syndrome or polycystic ovarian morphology?

**Design:**

Cross-sectional, case-control.

**Setting:**

Women who delivered preterm in 1999–2006. For each index woman, a woman with a term delivery was identified.

**Patients:**

Participation rate was 69%. Between 2006–2008, 262 women were included, and diagnosed to have polycystic ovary syndrome, polycystic ovarian morphology or to be normal controls.

**Intervention(s):**

Blood tests, a clinical examination and vaginal ultrasound.

**Main Outcome Measure(s):**

Anti-Mullerian Hormone / antral follicle count -ratio, SHBG, androstenedione and insulin, to test potential influence on the Anti-Mullerian Hormone / antral follicle count -ratio.

**Results:**

Mean Anti-Mullerian Hormone / antral follicle count ratio in women with polycystic ovary syndrome or polycystic ovarian morphology was similar to that of the controls (polycystic ovary syndrome: 1,2 p = 0,10 polycystic ovarian morphology: 1,2, p = 0,27 Controls 1,3). Anti-Mullerian Hormone showed a positive linear correlation to antral follicle count in all groups. Multivariate analysis did not change the results.

**Conclusions:**

We confirmed the positive correlation between AMH and follicle count. Anti-Mullerian Hormone seems to be a reliable predictor of antral follicle count, independent of polycystic ovary syndrome diagnosis or ovarian morphology.

## Introduction

Anti-Mullerian Hormone (AMH) is produced by the granulosa cells in the premature ovarian follicle. The level of AMH is relatively constant during the menstrual cycle as neither the primordial follicles, the dominant follicle nor corpus luteum secrete AMH [1–4]. During the menstrual cycle, the intra-individual variation in AMH may be up to 13% in infertile women with a regular cycle [5]. The intra-follicular concentration of AMH depends on the follicle size [6]. As AMH is mainly produced by the small antral follicles, it can be used as a proxy for the remnant follicle pool [7]. AMH peaks when women are in their early twenties [8]. Later in life, AMH decreases until menopause. In menopause it is no longer detectable [9].

Contrary to the normal physiological condition, where primordial follicles follow a continuous development, women with polycystic ovary syndrome (PCOS) have their ovarian follicles arrested in the pre-antral and antral stages [10]. At least two out of three criteria have to be fulfilled to meet the Rotterdam 2003 criteria for PCOS; oligo- or anovulation (OA), clinical and/or biochemical signs of hyperandrogenism (HA), and the presence of polycystic ovaries defined as at least 12 pre-antral follicles, 2–9 mm in diameter and/or increased ovarian volume >10 ml in at least one ovary (AFC) [11–14]. The cut-off at 9 mm seems crucial, as in vitro studies show that AMH-levels are low or undetectable in larger follicles [15].

Recently AMH-levels were shown to reflect polycystic ovarian morphology (PCOM), to a high extent. Although AMH cut-off levels of 10–20 pmol/l identifies PCOM (AFC ≥ 12) with a high sensitivity (resp. 98.8 and 91.6%), the corresponding specificities were poor (resp. 39.8 and 69.8%) [16]. A recent study found asymptomatic women with PCOM to have increased levels of AMH compared to asymptomatic women with normal ovaries [17]. When combined with other PCOS- criteria, the sensitivity and specificity in distinguishing women with PCOS from women with ovulatory cycles without HA, increases to 92% and 97%, respectively [18–19].

Even in follicles of similar size, in vitro ovarian cell cultures from women with PCOS show substantially higher levels of AMH as compared to women with normal cycles [15].

AMH-excess might in part be explained by increased AMH synthesis per follicle in PCOS-women, rather than reflecting the increased number of follicles arrested in the pre-antral and antral stages. Importantly, AMH is essential in limiting the further growth of pre-antral and antral follicles, possibly by inhibition of the effect of follicle stimulating hormone (FSH) [20]. There may be a subgroup of PCOS-women where follicles exceed the average production, as a recent study found the AMH/AFC ratio to be significantly increased in 87 PCOS women when compared to controls, while the AMH/AFC ratio in 131 PCOM-women was comparable to that of the control-group [21].

In the present study we aimed to analyze the relation between AMH and AFC in women with PCOS and PCOM and in women without these features, i.e. normal controls. To do so, we used data from a previously published case-control study [16].

## Methods

### Study Population

The present study comprised 262 women from a former case-control study [22]. In the original study women from a well-defined hospital catchment area (Namsos hospital, Norway) consisting of 17 municipalities in Nord-Trøndelag County were included. Women who had experienced a preterm birth were compared to women with a term birth after an uncomplicated pregnancy. Two-hundred and eighty-three out of 410 invited women responded, leading to a response rate of 69%. Twenty-one women were subsequently excluded because of a language barrier, or pregnancy/breast feeding at the time of inclusion. During former published results on preterm deliveries, women with twin deliveries in the index pregnancy were excluded (n = 21), but were included in the current study. In all, 133 of the 262 included women had a history of preterm birth. Clinical examination and blood drawing were performed between October 2006 and April 2008. S1 Fig shows the design of the former study, which was the basis of the current study.

Oligo-anovulation (OA) was defined as self-reported menstrual cycle length of ≥ 35 days or < 10 menstrual periods per year. Clinical hyperandrogenism (HA) was defined as a FG score ≥ 8 [23]. Biochemical HA was defined as serum testosterone > 2.5 nmol/l, free testosterone index (FTI) ≥ 0.6 and/or androstenedione (A4) ≥ 10.0 nmol/l.

PCOM was defined as ≥ 12 pre-antral follicles measuring 2–9 mm in diameter, and/or increased ovarian volume (>10 ml) in at least one ovary. PCOS was defined according to the Rotterdam criteria [11]. Based on the above mentioned criteria the study population was classified as PCOS (n = 56), PCOM (n = 58) and controls (n = 148). The phenotypic characteristics of the PCOS women were as follows: HA + PCOM (n = 27), HA + OA + PCOM (n = 16), OA + PCOM (n = 12) and HA + OA (n = 1). Accordingly, only one woman with PCOS did not have PCOM, and was reported among the 149 women without PCOM in the former publication [16]. Among the 148 controls, 16 had isolated HA and three had OA. All the 58 PCOM women were eumenorrhoic and normo-androgenic.

Seven women with missing AFC in either the left or the right ovary were censored in the subsequent analyses of AFC and AMH / AFC-ratio. Six women with AFC ranging from 4 to 17 belonging to the control group were included in the subsequent analyses, despite having their AMH-value measured to be zero.

In the original study, a glucose tolerance test was performed, as well as 3 measurements of blood pressure [16,22].

The Study was approved by the Regional Committee for Research Ethics in Health Region IV in Norway. An informed consent form was signed by all women before inclusion in the study. The study was carried out according to the Helsinki Declaration.

### Measurement of Antral Follicles

The gynecological examination was performed with US equipment General Electric Logiq Book XP with vaginal probe 7,5 MHz (General Electric Medical Systems, Solingen, Germany). Ovary size was measured in three dimensions, and the volume was subsequently calculated by the formula: height × width × depth × 0.5. All visible follicles of 2–9 mm in diameter in both ovaries were included in the AFC. All ultrasound examinations were performed by the same investigator (TBE).

### Laboratory Methods

Blood samples were drawn from an antecubital vein between 08 and 11 am after an overnight fast, centrifuged at room temperature within 30 minutes and stored at -70°C until analysis.

Although the intention was that the blood draw and clinical examination should be performed within the first 5 days of the menstrual cycle this aim was only achieved in 32 participants. S2 Fig shows the distribution of women with PCOS, PCOM and controls among quartiles of years in between the blood draw and the analysis of insulin, androgens and AMH.

For the AMH analysis, an enzymatically amplified two-site immunoassay (ACTIVE^®^MIS/AMH enzyme-linked immunosorbent assay (ELISA)) was used. Reagents and calibrators for the AMH analysis were supplied by the manufacturer (Diagnostic Systems Laboratories, Inc, Webster, TX, USA). For the A4 analysis, a competitive immunoassay using antibody-coated tubes was used (Coat-A-Count^®^). Reagents and calibrators for the A4 analysis was supplied by the manufacturer (Siemens Medical Solutions Diagnostics, Los Angeles, CA, USA), whereas ethyl ether was the organic solvent extraction used prior to quantification. For the testosterone analysis, an enzyme immunoassay for the quantitative determination in serum (ELISA) was used. Reagents and calibrators for the testosterone analysis was supplied by the manufacturer (DRG Instruments GmbH, Marburg, Germany), whereas dichloromethane was the organic solvent extraction used prior to quantification. Sex hormone binding globulin (SHBG) and insulin were measured quantitatively in serum using an ELISA method, with reagents and calibrators from the manufacturer (DRG Instruments GmbH, Marburg, Germany) [22]. FTI was calculated according to the formula; testosterone×10/SHBG.

All measurements were performed in singles, and all analyses were performed on kits from the same batch. Intra- and inter-assay coefficients of variation were 4.2% and 7.7% for AMH, 5.6% and 2.2% for A4, 9.5% and 14.0% for testosterone, 6.6% and 5.5% for SHBG and 3.6% and 4.9% for insulin.

### Statistics

All statistical procedures were performed using the PASW version 20 (IBM, SPSS, Armonk, NY, USA). Means and standard deviations (SD) were calculated, and the differences between the study groups were compared with two-tailed Mann-Whitney U tests for independent samples or Pearson’s chi-square test. Significance was set as a p-value < 0.05.

Univariate and multivariable linear regression analyses were used to study variables possibly associated to AMH. Variables with a p-value ≤ 0.1 in univariate analyses in at least one study group were included in the multivariable analyses.

## Results

### Population Characteristics

Women with PCOS and PCOM were significantly younger than controls, while body mass index (BMI) was comparable in the three groups (Table 1). A4 tended to be higher in PCOS women compared to PCOM women (p = 0.02), while in PCOM it was higher than in controls (p< 0.01). The FTI in PCOS-women was twice of that in PCOM-women and controls (p< 0.01). Insulin levels were increased in PCOS-women as compared to the control group. In PCOM-women, the mean insulin-level was similar to the control group.

**Table 1:** Clinical and biochemical characteristics according to study group.

	PCOS Mean (SD)	P-value*	PCOM Mean (SD)	P-value*	Controls Mean (SD)
Age (years)	33.3 (5.5)	<0.01	33.0 (4.6)	<0.01	36.2 (4.8)
BMI (kg/m^2^)	27.8 (5.7)	0.32	25.7 (4.4)	0.19	27.0 (5.2)
A4 (nmol/L)	6.4 (3.0)	<0.01	5.0 (1.7)	<0.01	4.4 (2.0)
FTI	0.4 (0.3)	<0.01	0.2 (0.1)	0.37	0.2 (0.1)
Insulin (pmol/L)	101.3 (54.1)	0.01	79.6 (34.5)	0.77	80.9 (35.9)
AMH (pmol/L)	44.8 (27.5)	<0.01	32.8 (20.4)	<0.01	14.6 (11.8)
AFC (no.)	35.7 (15.0)	<0.01	27.5 (7.0)	<0.01	12.5 (5.0)
AMH/AFC ratio	1.2 (0.5)	0.10	1.2 (0.6)	0.27	(1.4)

*Difference compared to controls; Mann Whitney U test for independent samples

PCOS: Polycystic Ovary Syndrome, PCOM: Polycystic ovarian Morphology, BMI: Body Mass Index, A4: Androstenedione, FTI: Free testosterone index, AMH: Anti Müllerian Hormone, AFC: Antral Follicle Count.

Analyzing data on AMH and AFC separately for women aged above or below the median age of 35 years, confirmed that AMH and AFC are increased in women with PCOS or PCOM as compared to control women (S1–S3 Tables).

The younger group (≥ 20 and ≤ 35 years of age) had higher mean AMH and AFC as compared to the elder group (> 35 years of age), and this pattern was seen in women with PCOS, PCOM and in controls, with the exception of mean AMH in PCOM-women, which was not different in the younger vs the elder group (S1–S3 Tables).

### AMH/AFC Ratio

**Fig 1:**
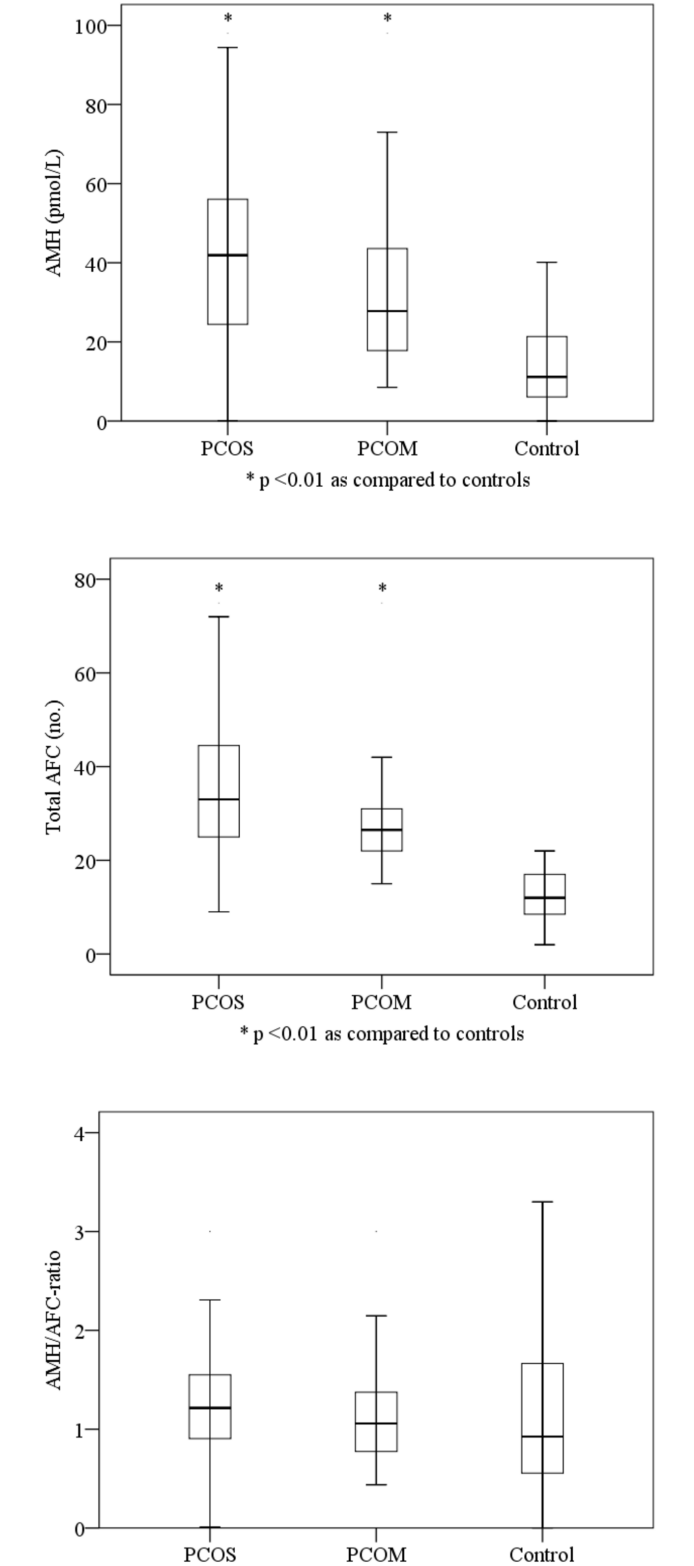
Boxplots of AMH, AFC and AMH/AFC-ratio in women with PCOS, PCOM and normal controls.

The AFC in women with PCOS was 3-fold increased, and in PCOM-women 2-fold increased compared to controls (p< 0.01). Also AMH was 3- and 2-fold increased in women with PCOS and PCOM compared to controls, resulting in comparable AMH/AFC-ratios; 1.2, 1.2 and 1.3 in PCOS, PCOM and controls (Table 1, Fig 1).

In young as well as elder women with either PCOS or PCOM, the AMH/AFC-ratio was not different from that of control-women (S1–S3 Tables).

**Fig 2:**
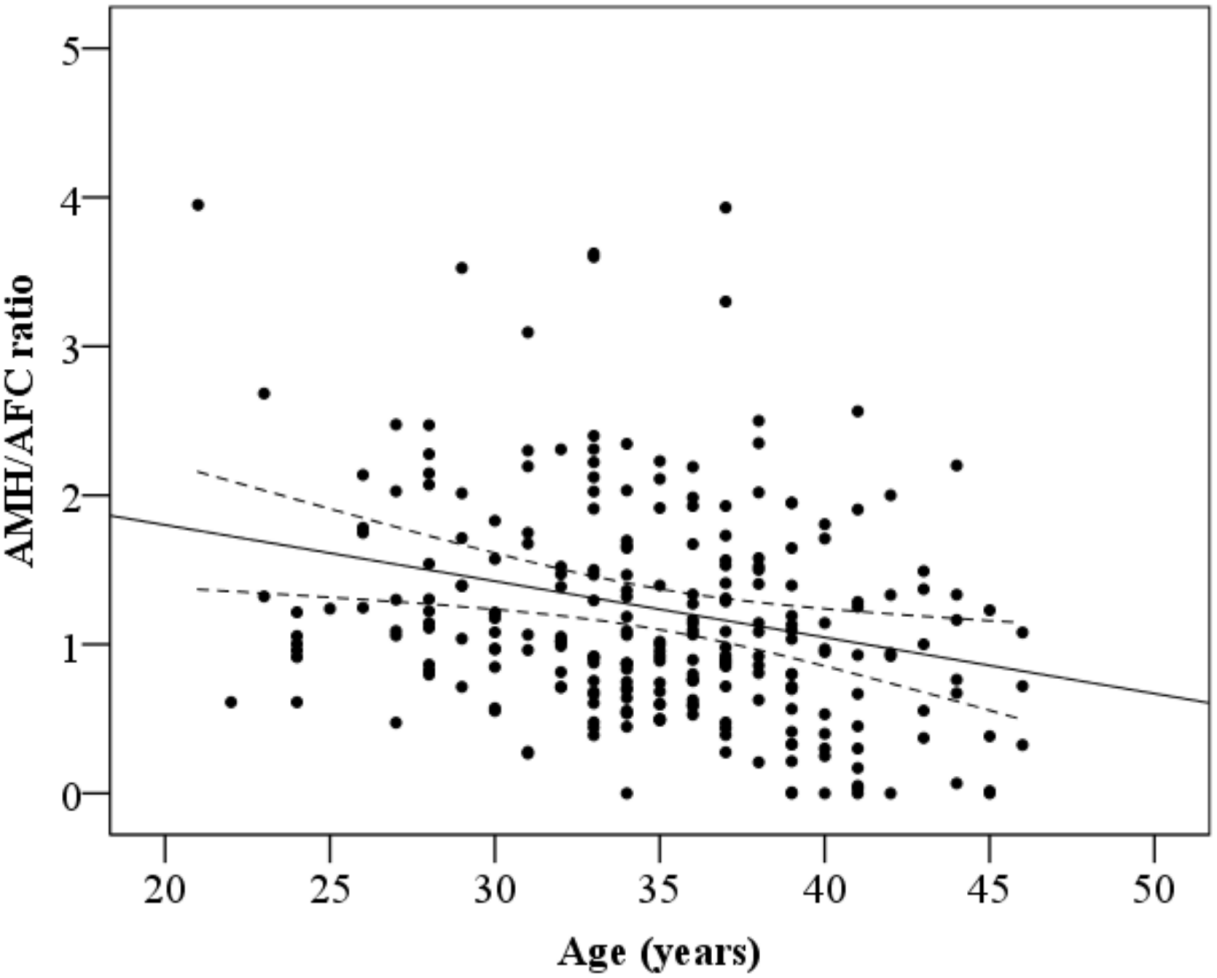
AMH/AFC-ratio according to age in 262 participants. * Censored and not shown 2 extreme values of AMH/AFC-ratio, which belonged to 2 control women, respectively 13.30 and 7.58, stipled line: 95% Confidence interval of the regression line.

The mean AMH/AFC-ratio in younger PCOS and PCOM-women (≥ 20 and ≤ 35 years of age) was not different from that in the elder group (> 35 years of age) (S1–S3 Tables, Fig 2).

The mean AMH/AFC-ratio was significantly higher in the young control group (≥ 20 and ≤ 35 years of age) as compared to the elder control-group (> 35 years of age) (S1–S3 Tables, Fig 2).

### Predictors of AMH

In univariate regression analyses, AMH decreased with age in all three study groups. A4 was positively correlated to AMH in PCOM only (Table 2). BMI, FTI and insulin levels showed no correlations to AMH in any of the three study groups. High AFC was strongly correlated to high AMH in all three study groups (PCOS: r = 0.77, PCOM: r = 0.70, normal controls: r = 0.42). The linear relationship between age and AMH/AFC-ratio is shown in Fig 2 (β: -0.04, CI95: -0.06; -0.01). This β expresses the lowering in AMH/AFC-ratio for each unit of rise in age. The linear relationships between AFC and AMH were as follows in the three study groups: PCOS: β: 1.42, CI95: 1.09; 1.74, PCOM: β: 2.04, CI95: 1.47; 2.61, normal controls: β: 0.99, CI95: 0.64; 1.35). These βs express the rise in AMH for each unit of rise in AFC.

**Table 2:** Regression analysis of AMH levels.

	PCOS (n = 56)			PCOM (n = 58)			Controls (n = 148)		
Variable	β	CI95	p	β	CI95	p	β	CI95	p
Univariate									
Age	- 1.47	- 2.78; -0.16	0.03	- 1.49	- 2.61; -0.37	0.01	- 0.82	- 1.19; -0.44	< 0.01
BMI	- 1.11	- 2.41; 0.18	0.09	- 0.62	- 1.86; 0.61	0.32	0.25	- 0.11; 0.62	0.18
A4	2.08	- 0.32; 4.48	0.09	5.06	2.13; 7.99	< 0.01	0.67	- 0.29; 1.64	0.17
FTI	10.89	-18.36; 40.14	0.46	-7.09	- 48.84; 34.65	0.73	6.21	-8.92; 21.34	0.42
Insulin	0.06	- 0.08; 0.20	0.37	- 0.10	- 0.26; 0.05	0.19	0.02	- 0.04; 0.07	0.53
AFC	1.42	1.09; 1.74	< 0.01	2.04	1.47; 2.61	< 0.01	0.99	0.64; 1.35	< 0.01
Multivariable									
Age	0.05	- 0.92; 1.03	0.91	- 0.55	- 1.44; 0.33	0.22	- 0.62	- 0.98; -0.26	< 0.01
BMI	- 1.09	- 1.97; -0.21	0.02	- 0.12	- 1.09; 0.85	0.81	0.31	- 0.02; 0.63	0.06
A4	1.05	- 0.59; 2.69	0.20	2.97	0.62; 5.31	0.01	0.45	- 0.42; 1.31	0.31
AFC	1.37	1.03; 1.72	<0.01	1.72	1.14; 2.31	< 0.01	0.87	0.52; 1.22	< 0.01

PCOS: Polycystic Ovary Syndrome, PCOM: Polycystic Ovary Morphology, β: Regression coefficient, CI95: Confidence interval of β, p: probability-value, A4: Androstenedione, FTI: Free Testosterone Index, AFC: Antral Follicle Count.

### Multivariable Regression

AFC remained positively correlated to AMH in all three groups also when we adjusted for age, BMI and A4. Age remained negatively correlated to AMH in control women only.

The tendency of a negative correlation between BMI and AMH was significant in PCOS-women. In normal control women, BMI tended to be positively correlated to AMH, although not reaching statistical significance.

### Hormonal Contraceptive Use

Twenty-one percent of the PCOS-women, 36% of the PCOM-women and 39% of the control women reported that they used hormonal contraceptives at the blood draw. Excluding women on hormonal contraception from the analyses, did not alter the results, i.e. the AMH/AFC-ratio remained similar in women with PCOS, PCOM and the controls.

Among those 90 patients who reported the use of hormonal contraception, 25 reported the use of oral hormonal contraception, and one woman reported the use of dermal contraception with progestin and estrogen. The distribution of the use of hormonal contraception among women with PCOS, PCOM and controls is reported in S4 Table.

Only 2 out of the 25 women who reported to use of oral contraception, did mention the brand of the oral contraception, and in both cases it was a progestin-only with a 3^rd^ generation gestagene.

### Blood Pressure, Impaired Glucosetolerance, and Smoking Status

There was no difference in neither systolic nor diastolic blood pressure (mean of last 2 of 3 measurements) when we compared the groups of PCOS, PCOM to the control group (S5 Table).

The prevalence of diabetes was 1.8% in the PCOS-group, 8.6% in the PCOM-group, and 1.4% in the control group (S6 Table).

The prevalence of impaired glucose tolerance was 21.4% in the PCOS-group, 15.5% in the PCOM-group, and 18.2% in the control group (S6 Table).

The prevalences of smoking at the blood draw among the 3 groups were as followed: PCOS: 21.4%, PCOM: 19.0%, Controls: 21.6% (Data not shown).

## Discussion

The most important finding of this study is the close and similar relationship between serum AMH and AFC in PCOS, PCOM and normal control women. This supports the view that increased serum AMH levels in PCOS are results of a higher number of antral follicles and not increased synthesis of AMH per follicle. Importantly, this relationship between AMH and AFC was unaffected by whether or not the women used hormonal contraception.

Some studies report higher intra-follicular levels of AMH in PCOS women compared to controls, indicating that AMH-excess could result from overactive follicles [15, 24]. However, high intra-follicular levels of AMH do not necessarily imply increased release of AMH from the follicles.

Intra-follicular AMH levels were found to be 75-fold higher in anovulatory PCOS-women compared to women with normal ovary morphology, when follicles were size matched. Intra-follicular AMH decreased with increasing follicle-size [15]. Although this indicates that follicles of women with PCOM synthetize more AMH than those of women with normal ovary morphology, this was a highly selected, small group with unmatched controls [15]. The circulating AMH levels were not reported.

In PCOS-women referred to routine laparoscopy or laparotomy, intra-follicular AMH-levels were 60-fold higher than serum AMH-levels [24]. Intra-follicular AMH levels in anovulatory PCOS women were 6-fold higher than in eumenorrhoic women. Serum levels of AMH was highly correlated to the intra-follicular levels in PCOS-women (r = 0.86), in contrast to eumennorhoic controls [24].

Our results are partly in line with a former study (n = 104) which found a correlation of serum-AMH to follicle count when follicles were 2–5 mm but not in follicles 6–9 mm [25]. The AMH/AFC-ratios were found to be similar in women with (n = 59) or without (n = 45) PCOS [25].

A larger study on 366 healthy eumenorroic women, aged 20–41 years reported a stronger correlation (r = 0.86) between AMH and AFC than we found in our control group, but underestimation of AMH was observed when AFC exceeded 20 follicles [26]. AFC correlated positively to AMH if follicle size was < 8 mm, whereas it correlated negatively beyond that limit. This may explain why we found a somewhat weaker linear relation between AMH and AFC (2–9 mm). Although it has been shown that primordial follicles also contain AMH, most of the AMH-production originate from antral follicles sized 5–8 mm [27, 28].

Our results are contradicted by a recent study which found the median AMH/AFC-ratio to be significantly increased in 87 women with PCOS as compared to 131 women with PCOM and 218 normal controls [21]. Similar to our study they defined PCOS according to the Rotterdam criteria and PCOM if an ovary had more than 12 follicles measuring 2–9 mm. Although they reported age as a median, their women with PCOS, PCOM and the controls, must have been approximately 2–4 year younger than our respective groups. The median AMH and AFC-count in our control group (11.2 and 12 respectively) are comparable to those of the control group of Bhide (11.95 and 12 respectively), despite that the blood test in Bhides study was not fixed to any day of the menstrual cycle, while our study aimed for a blood test within the first 5 days of the menstrual cycle (although only successful in a minority of the participants).

To explore if the difference in the two studies could be explained by the severity of PCOS, we performed an analysis restricting our PCOS-group to 16 PCOS-women with all three key features (HA, OA, PCOM) and still found the AMH/AFC ratio to be similar to that of controls (AMH/AFC-ratio PCOS = 1.1, p = 0.61). Nevertheless, the input of that study was totally different from ours, as their population was referred to a fertility clinic; our population was included with at least one pregnancy, which makes it probable that our population have lesser women with severe PCOS.

AMH decreased independently with age, in normal control women only [8]. The absence of this relation in women with PCOS or PCOM, could represent the longevity of antral follicles with undiminished production of AMH.

Our results show that high BMI is correlated with lower AMH in PCOS-women. This is contrary to our expectations, as high BMI, high AMH-levels and high AFC are common findings in PCOS. Nevertheless, in multivariable analysis, BMI did not change the relation between AFC and AMH, indicating that the AMH/AFC-ratio is independent of BMI.

### Strengths and Limitations of Our Study

In our study, women with PCOS and PCOM were significantly younger than the controls. We did not have the possibility to correct for antral follicles size distribution towards 2 mm or 9 mm. Our findings of a similar AMH/AFC-ratio between the groups can be explained if there is only a minor or no increase in serum-AMH when follicle-size increases from 2 to 9 mm. Alternatively, the relative distribution of follicle-size within the size range of 2–9 mm is similar between the three groups, whereas the AFC increases from controls towards PCOS-women.

In this study we did not do any measurements to exclude non-classical adrenal hyperplasia, which overall can be the cause of approximately 4% of the female patients with androgen excess [29]. However, in Norway congenital adrenal hyperplasia is a relatively rare condition compared to other populations with another genetic background. Based on clinical experience approximately 1% of women referred for possible PCOS have other causes of hyperandrogenism.

We did not perform any imaging studies to exclude the possibility of androgen-producing tumors, which overall can account for approximately 0.2% of the cases of androgen excess [29]

In Norway, approximately 90% of women in the fertile age give birth, and the selection of participants to this study included consecutive participants who gave preterm birth, and the subsequent participant who gave birth at term. This selection implies that we have more women with preterm birth than we would expect from for instance a random sample from the background population. The consecutive inclusion of women who gave birth at term is believed to be more representative for the background population in the fertile age. Although the prevalence of PCOS was higher in the group who delivered preterm, there was no difference in mean age, AMH, AFC, or AMH/AFC-ratio among those who delivered preterm or at term (S7 Table) [30].

The “mix” of the term and the preterm group, implies a higher prevalence of PCOS than in the background population, as we did not include all term births in the period when we assembled the patients with preterm birth, but is an advantage in the current study, as this increased the power without increasing the study to compare the AMH/AFC-ratio in women with PCOS, to those without.

Our study is medium sized and fairly representative for women of fertile age. Several of the women diagnosed with PCOS or PCOM were not aware of their status before they entered the study. Among a random sample of women, AMH-levels seem to have a good correlation to follicle count in normal women as well as in women with PCOS or PCOM.

### Conclusion

AMH seem to be a reliable predictor of AFC (2–9 mm), independent of PCOS diagnosis or ovarian morphology. Larger studies are needed to assess whether this is true for other populations and all subgroups of PCOS.

## Supporting Information

S1 Database (PDF)

S1 Fig Inclusion and exclusion decision tree for the current study. (DOCX)

S2 Fig Period from blood draw to analysis, in quartiles of the passed period. Black columns: Women with PCOS. Grey columns: Women with PCOM. White columns: Control women.
(DOCX)

S1 Table Young group 21–35 years (N = 140). Difference compared to controls; Mann Whitney U test for independent samples.
(DOCX)

S2 Table Elder group 36–46 years (N = 122). Difference compared to controls; Mann Whitney U test for independent samples.
(DOCX)

S3 Table Values from S1 and S2 tables: Young group versus elder group. Mann Whitney U test for independent samples.
(DOCX)

S4 Table Method of contraception in 90 women who used contraception with hormones. (DOCX)

S5 Table Mean systolic and diastolic blood pressure, mean of last 2 of 3 measurements. Difference compared to controls; Mann Whitney U test for independent samples.
(DOCX)

S6 Table The prevalence of diabetes, impaired glucose tolerance (IGT), and normal glucose tolerance (NGT) among women with PCOS, PCOM, and in controls. (DOCX)

S7 Table Mean age, AMH, AFC, AMH/AFC-ratio, and prevalence PCOS among those who delivered preterm and at term. Difference compared to controls; Mann Whitney U test for independent samples. Pearson’s chi-square test.
(DOCX)
