# Supplementary material for: Does AMH Reflect Follicle Number Similarly in Women with and without PCOS?
Source: PLoS One. 2016 Jan 22;11(1):e0146739. doi: 10.1371/journal.pone.0146739 (PMC4723054; doi:10.1371/journal.pone.0146739)
Supplement: S1 Database — (PDF) [file pone.0146739.s001.pdf]

Category	AMH	Age	BMI	A4
	pmol/L			nmol/L
Hyperandrogenism (HA)	13.2	33	38.40245	2.51
Normal control	20.2	38	26.29691	2.55
Normal control	7.2	46	23.42355	4.09
Normal control	30.4	33	22.79282	2.3
Hyperandrogenism (HA)	21.9	36	26.92378	10.5
Polycystic Ovarian Morphology (PCOM)	35.4	34	21.54051	6.91
Normal control	13.7	38	20.00297	2.37
Normal control	11.1	37	23.49792	3.53
Normal control	25.2	33	22.5537	4.78
Normal control	10.9	33	24.7641	5.13
Normal control	7.4	36	29.83235	2.1
Normal control	8.8	44	32.38836	4.33
Hyperandrogenism (HA)	19.8	37	24.50895	4.54
Polycystic Ovarian Morphology (PCOM)	50.1	37	22.19172	5.83
Normal control	2.6	43	23.79536	2.51
HA + PCOM	41.9	41	26.3608	6.81
Normal control	5.7	39	32.03787	6.91
Normal control	11.7	39	31.50352	4.64
HA + OA + PCOM	44.1	34	25.0995	6.39
HA + PCOM	20.6	38	25.76966	6.67
Normal control	10.9	33	23.62598	3.28
Polycystic Ovarian Morphology (PCOM)	61.6	33	27.12008	5.34
Normal control	16	44	28.7432	3.81
HA + PCOM	23.3	27	31.0608	11.3
Normal control	4.6	35	25.29263	5.17
Normal control	10.8	46	26.1012	2.97
Oligo-anovulation (OA)	8	42	24.60444	4.19
HA + OA + PCOM	43.9	35	42.19813	3.67
Polycystic Ovarian Morphology (PCOM)	18.9	38	22.1981	6.29
Polycystic Ovarian Morphology (PCOM)	46.1	34	24.84079	6.04
Hyperandrogenism (HA)	0.3	37	24.53894	2.93
Normal control	28.3	34	22.04006	6.25
OA + PCOM	41.9	31	19.31803	3.25
Polycystic Ovarian Morphology (PCOM)	24.9	26	26.12688	6.32
Polycystic Ovarian Morphology (PCOM)	85.5	33	23.02154	9.78
Normal control	7.6	37	42.93816	3.95
Normal control	55.7	31	26.85043	6.5
Normal control	39.9	34	39.75317	6.7
Normal control	6.1	34	32.61978	2.03
Normal control	19.8	27	22.80854	2.51
Hyperandrogenism (HA)	21.9	37	31.50826	9.08
HA + PCOM	53.7	43	25.35903	7.3
Normal control	2.7	41	20.31746	3
Normal control	9.6	37	30.14792	3.56
Normal control	14.7	37	24.17778	6.77
Polycystic Ovarian Morphology (PCOM)	32.2	28	21.4083	2.2
OA + PCOM	36.3	38	23.70323	4.96
Normal control	5.9	34	28.46963	4.68

Normal control	7.6	37	22.75976	3.32
HA + OA + PCOM	14.6	36	34.82094	13.7
Normal control	1.5	41	22.58955	2.13
Normal control	29.6	28	24.84295	3.74
Polycystic Ovarian Morphology (PCOM)	73	28	28.63613	5.1
Polycystic Ovarian Morphology (PCOM)	32.7	37	35.00111	4.78
Normal control	16.4	39	28.46648	7.96
Hyperandrogenism (HA)	28.2	41	28.4083	3.84
Normal control	32.5	40	22.91226	5.62
Normal control	8.3	36	31.68563	4.92
Normal control	18.4	40	20.82094	3.88
HA + PCOM	13.6	39	25.67493	2.62
HA + PCOM	20	32	29.69479	5.31
HA + OA	0.1	39	26.2259	2.62
Hyperandrogenism (HA)	13.9	34	24.33748	2.86
Polycystic Ovarian Morphology (PCOM)	17.9	39	23.62764	2.55
Polycystic Ovarian Morphology (PCOM)	17.6	39	22.57813	4.43
HA + PCOM	27.3	27	35.7674	5.83
Normal control	15.2	36	31.83615	8.31
Polycystic Ovarian Morphology (PCOM)	16.1	35	29.91	5.66
OA + PCOM	55.4	33	31.60814	6.88
Polycystic Ovarian Morphology (PCOM)	36.6	28	20.76144	4.82
Polycystic Ovarian Morphology (PCOM)	21	38	33.48948	3.14
OA + PCOM	120	32	18.53778	5.87
Normal control	30.8	28	19.10552	3.35
Normal control	0	41	25.13918	2.3
HA + PCOM	60.7	34	31.5917	6.63
HA + PCOM	40.8	36	24.72261	5.45
Normal control	10.1	34	21.50756	6.91
Polycystic Ovarian Morphology (PCOM)	27.2	30	34.60515	3.07
OA + PCOM	51.2	27	22.76346	6.53
Normal control	51.1	37	31.03022	3.14
Normal control	22	34	28.47001	5.59
Normal control	7.4	44	21.12548	2.83
Normal control	19.9	40	24.62041	5.1
Normal control	0	34	23.93186	3
Oligo-anovulation (OA)	7.4	33	20.72144	2.27
Normal control	9.5	24	20.17389	2.1
Normal control	8	38	29.8449	3.74
Polycystic Ovarian Morphology (PCOM)	45.6	30	29.29055	5.31
Normal control	17.1	36	34.99588	2.55
Polycystic Ovarian Morphology (PCOM)	28	30	24.95796	4.23
Normal control	0	42	21.82561	2.58
Polycystic Ovarian Morphology (PCOM)	17.7	37	25.73402	4.19
Normal control	4.3	33	24.09552	1.68
HA + OA + PCOM	57.7	31	21.13058	4.47
HA + PCOM	22.1	36	24.93588	3
Normal control	11.2	35	22.62569	2.55
HA + PCOM	48.2	33	23.46041	5.31
Polycystic Ovarian Morphology (PCOM)	26.9	34	30.3288	3.14

Normal control	6.6	28	31.73993	3.42
Polycystic Ovarian Morphology (PCOM)	11.9	35	27.10656	2.13
Normal control	28.2	29	23.32947	2.9
Normal control	27	37	22.74532	2.27
Polycystic Ovarian Morphology (PCOM)	27.9	31	24.88188	7.26
Normal control	18	33	22.16309	5.27
Normal control	12.3	35	26.60038	2.58
OA + PCOM	39.4	31	30.0479	1.89
Normal control	0	39	25.8951	1.71
Normal control	10.5	34	19.29204	5.83
Normal control	6.1	43	24.09552	3.88
Normal control	11.1	34	24.79571	2.51
Normal control	15.1	33	25.43269	2.86
Polycystic Ovarian Morphology (PCOM)	20.2	34	27.2903	4.09
Normal control	0.4	41	31.22299	3.7
HA + PCOM	53.1	33	23.7338	5.62
Polycystic Ovarian Morphology (PCOM)	33.4	41	21.57702	3.77
OA + PCOM	82.9	38	28.46737	3.42
HA + OA + PCOM	35.2	29	40.62509	7.12
HA + OA + PCOM	66.2	32	28.65052	9.71
Normal control	12.2	34	34.88281	3.35
Normal control	2.3	38	26.14819	3.14
Normal control	0.1	45	27.80015	4.37
Normal control	18.1	33	28.71321	4.05
Normal control	0	42	35.26754	3.42
Normal control	0	45	21.90828	2.9
Polycystic Ovarian Morphology (PCOM)	45.8	30	27.09343	4.92
Normal control	1.7	41	25.26158	3.18
Polycystic Ovarian Morphology (PCOM)	32.2	25	26.08013	4.64
Hyperandrogenism (HA)	6.1	34	32.7574	6.22
OA + PCOM	13.8	40	29.0688	1.96
Polycystic Ovarian Morphology (PCOM)	31.5	32	23.38	5.76
Normal control	32.5	40	25.89994	3.63
Normal control	29.4	37	40.24321	3.21
Normal control	8.4	33	25.84777	2.62
Hyperandrogenism (HA)	36.5	27	21.81302	2.58
Polycystic Ovarian Morphology (PCOM)	15.3	22	17.67052	4.26
OA + PCOM	56.3	32	31.1695	2.34
Normal control	4.2	39	21.01641	2.58
Normal control	14.4	33	34.36638	6.29
Normal control	40.1	35	29.45822	4.37
HA + PCOM	141	36	31.2472	9.11
Normal control	32.5	38	30.67368	4.33
HA + OA + PCOM	44.8	39	28.08433	6.81
Polycystic Ovarian Morphology (PCOM)	26.9	35	26.10917	8.31
Polycystic Ovarian Morphology (PCOM)	57.8	37	23.98664	5.97
Normal control	2.5	40	18.74556	5.62
Normal control	32.9	31	22.86469	4.05
Normal control	28.2	29	23.09154	2.72
Polycystic Ovarian Morphology (PCOM)	27.4	39	18.81374	6.32

HA + PCOM	92.5	29	23.59799	12.6
Polycystic Ovarian Morphology (PCOM)	9.2	37	35.14335	4.19
HA + PCOM	51.7	26	21.30428	8.45
HA + PCOM	50.8	28	19.66904	4.3
Hyperandrogenism (HA)	2.4	36	29.90411	10.9
Normal control	2.7	34	24.74745	7.47
Normal control	6.5	41	24.72656	4.85
Polycystic Ovarian Morphology (PCOM)	27.9	30	23.86425	4.54
Polycystic Ovarian Morphology (PCOM)	32.1	35	26.20307	4.57
Polycystic Ovarian Morphology (PCOM)	56.7	30	26.35751	2.34
Normal control	8.8	39	24.12381	4.75
Polycystic Ovarian Morphology (PCOM)	99.3	23	19.78015	6.88
Normal control	1.9	34	27.52302	8.24
Normal control	12.2	37	26.70782	8.1
HA + PCOM	55.8	29	25.57257	10.2
Polycystic Ovarian Morphology (PCOM)	14.7	41	23.04192	1.5
Normal control	1.6	31	24.37128	2.23
Normal control	9.3	33	25.12397	4.19
Normal control	37.6	38	18.81701	8.87
Normal control	24.9	35	35.23834	3.42
Normal control	0.1	41	36.69589	3.28
HA + PCOM	39	38	24.3204	5.83
Normal control	25.3	31	29.21875	2.27
HA + PCOM	25.9	24	29.24211	11
Normal control	17.5	34	26.49492	4.57
Normal control	4.2	40	27.77778	2.44
Polycystic Ovarian Morphology (PCOM)	9.5	31	18.79376	6.01
Polycystic Ovarian Morphology (PCOM)	8.5	35	22.79282	3.14
Hyperandrogenism (HA)	44.6	35	36.40865	4.33
HA + OA + PCOM	94.4	26	21.57112	6.32
HA + PCOM	50.7	43	22.25747	4.61
Normal control	10.3	30	31.27309	4.78
HA + PCOM	73.6	36	27.51148	5.73
Polycystic Ovarian Morphology (PCOM)	55.9	36	28.51415	5.9
Normal control	19.1	33	25.75148	4.78
Normal control	26.5	39	31.17288	9.67
Normal control	8.1	37	24.34867	6.56
Normal control	20	33	25.85249	7.82
Polycystic Ovarian Morphology (PCOM)	54.3	29	25.36332	5.2
Normal control	25.8	34	28.23329	4.78
Normal control	2.6	46	28.08163	4.09
HA + PCOM	40.7	36	21.03482	10.8
Normal control	11.2	32	24.70939	5.87
Normal control	9.8	24	22.73298	9.39
Normal control	12.1	32	29.01786	4.23
HA + PCOM	57.4	28	31.03294	5.94
Normal control	9.4	42	22.49691	3.67
Normal control	2.2	37	30.13528	7.33
Normal control	5	29	32.00609	6.04
HA + OA + PCOM	20.7	36	27.97951	7.51

Polycystic Ovarian Morphology (PCOM)	27.7	36	28.55469	3.7
OA + PCOM	85.5	26	24.69041	7.19
HA + PCOM	28.1	24	41.80914	13.2
Normal control	31.6	21	30.14244	5.27
Polycystic Ovarian Morphology (PCOM)	23	36	26.00775	7.16
HA + PCOM	25.6	44	28.35992	10.8
Normal control	30.6	37	46.73136	3.56
Normal control	0	40	24.72506	4.54
Normal control	8	40	21.19011	4.26
Normal control	8.5	34	39.06609	2.86
Polycystic Ovarian Morphology (PCOM)	17.8	35	31.2016	6.56
Polycystic Ovarian Morphology (PCOM)	87	28	24.33748	7.4
HA + OA + PCOM	72.2	24	35.28341	11.7
Polycystic Ovarian Morphology (PCOM)	11.1	36	19.26531	3
HA + OA + PCOM	23.3	36	32.19908	3.56
Normal control	8.5	34	19.25447	6.18
Polycystic Ovarian Morphology (PCOM)	11.6	30	28.58543	5.27
Normal control	20.2	34	27.88965	4.16
Hyperandrogenism (HA)	17.3	42	36.80841	2.62
Polycystic Ovarian Morphology (PCOM)	29.8	32	21.84798	7.79
Normal control	16.1	30	24.60444	5.69
Normal control	2.3	45	20.53019	4.33
Normal control	20.9	45	27.7408	4.37
Normal control	11.4	32	27.47795	3.04
HA + PCOM	9.3	35	32.72	2.93
OA + PCOM	26.7	32	20.74219	3.74
Polycystic Ovarian Morphology (PCOM)	43.6	23	22.03164	4.78
HA + PCOM	28.5	38	20.64886	9.6
Normal control	22.7	36	30.63134	8.14
Normal control	6.1	44	27.51487	3.21
Oligo-anovulation (OA)	18.1	39	22.48267	3.95
Normal control	15.7	30	27.2284	9.01
Polycystic Ovarian Morphology (PCOM)	43	32	22.7048	4.82
HA + OA + PCOM	58.7	28	32.23239	6.01
Normal control	24.7	39	23.16774	4.5
Normal control	12.9	42	38.43694	1.22
Polycystic Ovarian Morphology (PCOM)	14.1	35	24.27093	5.9
Normal control	4.6	39	22.33469	6.08
Hyperandrogenism (HA)	37.9	38	37.14255	5.83
HA + OA + PCOM	55.3	28	24.6073	8.8
Normal control	19.6	34	24.38635	4.85
Hyperandrogenism (HA)	4.7	37	32.61708	3.53
Normal control	51.9	28	22.16067	3.35
Normal control	3	39	27.57795	4.12
Normal control	21.8	29	22.41027	2.17
Normal control	3.9	31	21.34066	2.34
OA + PCOM	23	33	23.05176	3.6
Polycystic Ovarian Morphology (PCOM)	53.3	34	24.84451	7.12
Polycystic Ovarian Morphology (PCOM)	37.8	32	28.6407	4.75
Normal control	6.2	39	23.44838	6.29

Polycystic Ovarian Morphology (PCOM)	9.4	38	32.8219	3
HA + OA + PCOM	0.6	44	34.53776	3.46
Normal control	22.5	41	25.39411	4.57
Polycystic Ovarian Morphology (PCOM)	21.2	28	37.6086	4.75
Hyperandrogenism (HA)	12.8	33	22.97778	4.4
Normal control	23.8	30	27.78547	6.39
Polycystic Ovarian Morphology (PCOM)	23.8	39	29.47846	2.27
Hyperandrogenism (HA)	4.4	40	32.95645	6.63
Polycystic Ovarian Morphology (PCOM)	24.7	37	25.67445	4.19
HA + OA + PCOM	7.9	39	34.88372	2.23
Normal control	10.8	35	26.77593	8.07
Polycystic Ovarian Morphology (PCOM)	9	27	22.22656	6.11
Normal control	9	43	26.01085	2.9
HA + OA + PCOM	41.3	24	34.72088	5.94

FTI	INSULIN	AFC	RATIO	CONTRACEPTION
	pmol/L			
0.266145	182.03	9	1.466667	No
0.314706	55.13	10	2.02	Yes
0.135	51.04	10	0.72	No
0.021944	56.46	15	2.026667	Yes
0.445434	122.54	10	2.19	No
0.15	88.7	26	1.361538	No
0.060976	58.34	9	1.522222	No
0.305882	64.88	12	0.925	No
0.110331	45.96	7	3.6	No
0.092982	50.25	16	0.68125	Yes
0.141623	33.61	14	0.528571	Yes
0.374603	79.14	4	2.2	No
0.298611	86.51	6	3.3	No
0.143876	47	32	1.565625	No
0.081839	80.76	7	0.371429	Yes
0.497561	125.2	22	1.904545	No
0.548223	103.56	8	0.7125	Yes
0.137283	137.49	6	1.95	No
0.328713	85.56	26	1.696154	No
0.360691	65.23	19	1.084211	Yes
0.141123	56.46	18	0.605556	No
0.198966	64.17	17	3.623529	No
0.092	88.21	12	1.333333	No
0.802326	111.15	22	1.059091	Yes
0.306931	59.45	5	0.92	No
0.10771	49.04	10	1.08	No
0.166667	60.55	4	2	No
0.260504	179.92	46	0.954348	No
0.387464	66.64	22	0.859091	Yes
0.144186	70.45	28	1.646429	No
0.144424	118.59	#NULL!	#NULL!	Yes
0.291845	56.84	17	1.664706	No
0.06729	57.96	25	1.676	No
0.542683	99.69	20	1.245	Yes
0.442073	51.83	37	2.310811	No
0.322946	159.84	16	0.475	Yes
0.333333	84.27	18	3.094444	No
0.253041	76.51	3	13.3	Yes
0.154839	61.28	3	2.033333	No
0.159341	38.33	8	2.475	Yes
0.544747	87.14	17	1.288235	No
0.280374	86.82	36	1.491667	No
0.098418	31.12	6	0.45	Yes
0.245562	109.42	11	0.872727	Yes
0.34903	61.28	15	0.98	Yes
0.127771	75.52	29	1.110345	Yes
0.221705	60.92	23	1.578261	No
0.176744	77.17	11	0.536364	No

0.112057	41.91	7	1.085714	No
0.798742	148.88	19	0.768421	No
0.132948	54.36	5	0.3	Yes
0.157277	83.66	13	2.276923	Yes
0.128689	94.87	34	2.147059	Yes
0.091589	138.45	25	1.308	Yes
0.308725	75.52	15	1.093333	Yes
0.129915	96.69	11	2.563636	No
0.124498	66.29	18	1.805556	Yes
0.384211	140.31	11	0.754545	No
0.044218	52.62	19	0.968421	No
0.082353	35.54	24	0.566667	No
0.217241	102.97	28	0.714286	No
0.248768	39.69	16	0.00625	No
0.110769	35.06	16	0.86875	No
0.044211	55.7	25	0.716	Yes
0.139601	73.51	22	0.8	No
0.617391	118.59	21	1.3	No
0.315534	98.5	20	0.76	Yes
0.3125	104.74	27	0.596296	Yes
0.309609	133.1	29	1.910345	Yes
0.066499	60.19	32	1.14375	Yes
0.218667	110.77	26	0.807692	No
0.340323	83.9	52	2.307692	Yes
0.085714	32.3	20	1.54	Yes
0.119822	95.02	12	0	Yes
0.529412	155.09	46	1.319565	Yes
0.271429	87.45	35	1.165714	No
0.098519	51.39	#NULL!	#NULL!	No
0.398126	174.99	28	0.971429	Yes
0.429012	81.67	47	1.089362	No
0.209591	65.83	13	3.930769	No
0.274157	93.88	15	1.466667	Yes
0.092382	54.75	11	0.672727	No
0.127379	101.74	21	0.947619	No
0.186242	45.64	4	0	No
0.040513	61.59	19	0.389474	No
0.023881	48.06	9	1.055556	Yes
0.340741	113.83	7	1.142857	Yes
0.315	75.73	29	1.572414	Yes
0.109116	77.85	15	1.14	No
0.117221	47.9	29	0.965517	No
0.163507	57.01	#NULL!	#NULL!	No
0.39418	64.68	20	0.885	Yes
0.142259	136.36	9	0.477778	Yes
0.33711	59.22	33	1.748485	No
0.224422	55.17	37	0.597297	No
0.029032	111.08	11	1.018182	Yes
0.267581	45.43	55	0.876364	No
0.1378	52.24	42	0.640476	No

0.236328	58.82	8	0.825	No
0.108453	74.66	24	0.495833	No
0.088462	63.92	14	2.014286	Yes
0.046632	89.78	14	1.928571	No
0.106977	63.53	29	0.962069	No
0.321656	62.56	12	1.5	Yes
0.154192	70.67	18	0.683333	No
0.210243	103.94	37	1.064865	No
0.205074	55.77	17	0	No
0.136719	54.75	12	0.875	No
0.082955	47.01	11	0.554545	No
0.046277	93.54	16	0.69375	Yes
0.085586	43.36	20	0.755	No
0.168203	47.23	27	0.748148	No
0.382979	87.78	12	0.033333	Yes
0.257541	54.75	41	1.295122	No
0.138042	50.53	26	1.284615	No
0.099684	119.27	59	1.405085	No
1.19337	206.91	#NULL!	#NULL!	Yes
0.977778	255.01	45	1.471111	No
0.184783	97.6	14	0.871429	Yes
0.045806	62.17	11	0.209091	Yes
0.19708	94.37	7	0.014286	Yes
0.06506	74.3	20	0.905	Yes
0.205941	55.37	5	0	No
0.137931	53.08	17	0	No
0.088679	77.14	39	1.174359	No
0.131148	50.96	10	0.17	No
0.214646	93.72	26	1.238462	No
0.275964	120.47	11	0.554545	No
0	121.07	26	0.530769	Yes
0.115476	55.58	31	1.016129	No
0.113861	96.63	19	1.710526	No
0.088496	83.73	17	1.729412	No
0.077156	58.2	19	0.442105	No
0.021084	46.56	18	2.027778	No
0.186813	83.31	25	0.612	No
0.249097	76.57	37	1.521622	No
0.273279	33.61	6	0.7	No
0.270793	97.49	6	2.4	Yes
0.198319	99.15	19	2.110526	No
0.522388	142.49	71	1.985915	No
0.210756	101.92	13	2.5	No
0.400881	105.63	23	1.947826	No
0.239688	98.98	27	0.996296	No
0.235152	62.06	41	1.409756	No
0.326797	51.95	10	0.25	Yes
0.147679	70.95	15	2.193333	No
0.309353	61.54	8	3.525	Yes
0.474006	45.69	23	1.191304	No

0.355609	62.06	54	1.712963	No
0.549505	133.92	21	0.438095	No
0.747368	106.22	29	1.782759	Yes
0.150461	63.1	39	1.302564	No
0.202335	102.64	3	0.8	No
0.357282	101.55	5	0.54	Yes
0.326582	89.8	7	0.928571	Yes
0.411765	127.63	23	1.213043	No
0.334177	103	23	1.395652	No
0.421488	74.59	31	1.829032	Yes
0.126147	77.76	11	0.8	No
0.158859	77.76	37	2.683784	No
0.284072	59.4	#NULL!	#NULL!	No
0.320175	128.9	17	0.717647	Yes
0.420635	86.6	40	1.395	No
0.065887	58.32	22	0.668182	No
0.185535	114.86	6	0.266667	Yes
0.156682	72.81	14	0.664286	Yes
0.189355	68.57	16	2.35	No
0.153409	98.41	13	1.915385	No
0.335244	99.72	2	0.05	No
0.224888	66.13	26	1.5	Yes
0.103131	95.17	11	2.3	No
0.423963	144.9	27	0.959259	No
0.24595	80.78	21	0.833333	No
0.225564	106.4	14	0.3	Yes
0.195011	69.29	#NULL!	#NULL!	No
0.14717	48.3	17	0.5	No
0.741935	121.5	20	2.23	Yes
0.183486	45.69	54	1.748148	No
0.165049	54.38	37	1.37027	No
0.473988	65.63	18	0.572222	Yes
0.169283	71.65	44	1.672727	No
0.298413	62.06	29	1.927586	Yes
0.291883	88.21	9	2.122222	No
0.472727	114.01	19	1.394737	No
0.190789	122.81	9	0.9	No
0.223022	58.32	9	2.222222	No
0.149254	85.79	39	1.392308	Yes
0.192635	52.02	11	2.345455	No
0.102586	44.33	8	0.325	No
0.278788	101.55	32	1.271875	No
0.137736	66.62	11	1.018182	No
0.339623	69.05	16	0.6125	Yes
0.14	62.06	17	0.711765	No
0.702381	143.7	72	0.797222	Yes
0.044737	82.05	10	0.94	No
0.138587	52.62	8	0.275	Yes
0.143403	98.04	7	0.714286	Yes
0.225191	109.72	33	0.627273	No

0.327711	51.79	26	1.065385	No
0.206086	69.65	40	2.1375	No
1.008734	194.29	28	1.003571	No
0.351464	123.11	8	3.95	Yes
0.48	102	21	1.095238	Yes
0.575693	111.33	22	1.163636	Yes
0.328671	218.7	20	1.53	No
0.283721	53.36	6	0	No
0.123482	101.2	7	1.142857	No
0.230337	67.84	12	0.708333	No
0.122695	87.16	20	0.89	No
0.193269	47.17	42	2.071429	Yes
0.720867	185.21	79	0.913924	No
0.186691	50.18	19	0.584211	Yes
0.232984	63.01	26	0.896154	No
0.092754	86.69	19	0.447368	No
0.33	106.34	21	0.552381	No
0.080935	86.5	19	1.063158	Yes
0.177305	283.41	13	1.330769	Yes
0.255639	39.12	29	1.027586	Yes
0.252019	92.34	19	0.847368	No
0.118487	45.79	6	0.383333	No
0.29771	67.84	17	1.229412	No
0.274627	106.79	14	0.814286	Yes
0.135272	86.05	19	0.489474	No
0.149909	34.13	27	0.988889	Yes
0.088462	61.03	33	1.321212	Yes
0.265501	56.12	31	0.919355	No
0.477212	133.99	17	1.335294	No
0.218263	76.84	8	0.7625	No
0.16358	76.36	16	1.13125	Yes
0.304069	54.91	13	1.207692	Yes
0.142655	93.82	31	1.387097	Yes
0.286467	305.53	48	1.222917	No
0.258427	40.68	15	1.646667	Yes
0.169533	125.96	14	0.921429	No
0.313808	41.81	19	0.742105	No
0.113725	43.66	14	0.328571	No
0.122368	104.77	5	7.58	No
0.353818	75.34	64	0.864062	No
0.173697	39.12	18	1.088889	No
0.694611	136.19	12	0.391667	Yes
0.167577	57.31	21	2.471429	No
0.316794	134.33	14	0.214286	No
0.022901	80.14	21	1.038095	Yes
0.037984	72.93	14	0.278571	No
0.073282	36.23	25	0.92	No
0.185668	47.85	45	1.184444	Yes
0.170388	88.69	36	1.05	No
0.173554	48.61	15	0.413333	No

0.109705	100	15	0.626667	No
0.17767	86.5	9	0.066667	No
0.131723	43.58	18	1.25	No
0.322344	233.14	#NULL!	#NULL!	No
0.288835	74.41	14	0.914286	No
0.316397	54.91	22	1.081818	Yes
0.03631	56.12	23	1.034783	No
0.290476	170.13	11	0.4	No
0.089209	122.03	29	0.851724	No
0.067831	115.25	24	0.329167	No
0.102013	90.85	18	0.6	No
0.240793	77.31	19	0.473684	No
0.228916	67.56	9	1	No
0.130097	93.4	34	1.214706	No

TYPE	Time blood	SBT	DBT	smoking
	Years			
	2.283368	163	101	No
progestin-only intrauterine device	2.264203	128.5	82.5	No
	2.264203	110	68.5	No
oral hormonal contraception	2.247775	123.5	66.5	No
	2.261465	112.5	71	Yes
	2.261465	109	70.5	No
	2.247775	90.5	55	No
	2.247775	122.5	68	No
	2.245038	101	64	No
oral hormonal contraception	2.245038	119.5	66	No
injectable progestin-only preparations	2.245038	126	79.5	No
	2.234086	124.5	76.5	No
	2.225873	145.5	80.5	No
	2.225873	100.5	58.5	No
oral hormonal contraception	2.223135	125	74	No
	2.223135	130	76.5	No
progestin-only intrauterine device	2.223135	135.5	74	No
	1.979466	169.5	103	Yes
	2.212183	120	67	Yes
progestin-only intrauterine device	2.209446	109	64	Yes
	2.209446	110	64.5	No
	2.190281	128	74.5	No
	2.190281	126	82	No
injectable progestin-only preparations	2.190281	118	69	Yes
	2.184805	114	67	Yes
	2.168378	119	68	No
	2.151951	108	65.5	No
	2.151951	140	79.5	No
injectable progestin-only preparations	1.979466	111	79.5	Yes
	2.017796	94.5	61	No
injectable progestin-only preparations	2.017796	124	73.5	Yes
	1.330595	117	71	Yes
	1.998631	101.5	56	No
progestin-only hormonal implants	1.982204	118	68	Yes
	1.982204	116.5	75.5	No
progestin-only intrauterine device	1.979466	127	80.5	No
	1.979466	121	84	No
progestin-only intrauterine device	1.976728	143	91.5	Yes
	1.976728	120.5	78.5	No
progestin-only intrauterine device	1.979466	126.5	74.5	No
	1.960301	129	77	No
	1.960301	122.5	62	No
injectable progestin-only preparations	1.960301	119.5	72.5	Yes
progestin-only intrauterine device	1.960301	125	79.5	No
progestin-only intrauterine device	1.957563	123.5	72.5	No
oral hormonal contraception	1.957563	113	59.5	No
	1.957563	110.5	73.5	No
	1.900068	123.5	75.5	No

	1.943874	121	76	No
	1.943874	129	78	No
oral hormonal contraception	1.943874	118.5	69.5	No
progestin-only intrauterine device	1.908282	119.5	70	No
oral hormonal contraception	1.908282	122	68	Yes
oral hormonal contraception	1.908282	129	66.5	No
progestin-only intrauterine device	1.905544	132	79.5	No
	1.905544	120	74.5	No
progestin-only intrauterine device	1.905544	127.5	78.5	No
	1.902806	121.5	62	Yes
	1.902806	112	71.5	No
	1.902806	106.5	67.5	No
	1.900068	121.5	73	No
	1.900068	111	73	No
	1.883641	111.5	63.5	No
progestin-only intrauterine device	1.883641	123.5	74.5	No
	1.883641	116	65	No
	1.867214	145	103.5	Yes
injectable progestin-only preparations	1.867214	117	68	No
progestin-only intrauterine device	1.864476	113	62	No
injectable progestin-only preparations	1.864476	133.5	85.5	No
oral hormonal contraception	1.861739	115	70.5	No
	1.785079	115	81	No
oral hormonal contraception	1.850787	120	74	No
oral hormonal contraception	1.850787	112.5	62.5	Yes
progestin-only intrauterine device	1.850787	117	69	Yes
progestin-only intrauterine device	1.785079	129.5	93.5	No
	1.842574	116	67	No
	1.806982	120.5	90	No
injectable progestin-only preparations	1.806982	116.5	80.5	No
	1.806982	111	60.5	No
	1.771389	116	67	Yes
progestin-only intrauterine device	1.804244	126	76.5	No
	1.804244	99.5	66	No
	1.804244	114	64.5	No
	1.787817	113	69	No
	1.787817	124.5	70	No
dermal contraception with progestin and	1.768652	116.5	60.5	Yes
progestin-only intrauterine device	1.785079	120.5	69	Yes
progestin-only intrauterine device	1.785079	126	68.5	No
	1.69473	137	81	Yes
	1.768652	114.5	62.5	No
	1.768652	99.5	60.5	No
oral hormonal contraception	1.768652	151	104	No
injectable progestin-only preparations	1.754962	130	77.5	No
	1.749487	110.5	67.5	No
	1.749487	108.5	69.5	No
oral hormonal contraception	1.749487	138	82	No
	1.746749	118	69	No
	1.292266	120.5	70.5	No

	1.69473	112	62	Yes
	1.40178	117	75	Yes
oral hormonal contraception	1.730322	107	61	No
	1.730322	116	67.5	No
	1.727584	109.5	64	No
progestin-only intrauterine device	1.727584	113	72	No
	1.730322	126	66.5	No
	1.713895	99	55.5	No
	1.711157	142	97.5	No
	1.727584	125	74.5	No
	1.689254	114	60.5	No
oral hormonal contraception	1.689254	122.5	71.5	No
	1.675565	121.5	68	No
	1.675565	126	78.5	Yes
progestin-only intrauterine device	1.675565	140	79.5	Yes
	1.650924	114	60.5	No
	1.650924	122	84	No
	1.637235	110	72	No
progestin-only intrauterine device	1.270363	112.5	64	No
	1.615332	160.5	88	No
oral hormonal contraception	1.059548	126	69	No
oral hormonal contraception	1.634497	121	72	No
progestin-only intrauterine device	1.634497	127.5	78	No
oral hormonal contraception	1.631759	122.5	72.5	No
	1.620808	142	91.5	No
	1.366188	112	62.5	Yes
	1.620808	109	69.5	No
	1.596167	114	74	No
	1.407255	128	85	No
	1.596167	129.5	89	No
progestin-only intrauterine device	1.407255	140	92.5	No
	1.407255	118.5	74	No
	1.32512	110	62.5	No
	1.368925	131.5	77.5	No
	1.368925	114	79	No
	1.36345	106.5	60	No
	1.347023	107.5	61	Yes
	1.347023	125	86.5	No
	1.344285	117	60.5	No
progestin-only intrauterine device	1.344285	114	64	No
	1.330595	131	90.5	No
	1.32512	132.5	85	No
	1.32512	105	61	Yes
	1.330595	112.5	70	No
	1.292266	118	77	No
	1.292266	93.5	64.5	No
progestin-only intrauterine device	1.289528	119	75	No
	1.289528	122	76	Yes
progestin-only intrauterine device	1.289528	107	68.5	No
	1.28679	132.5	91	No

	1.28679	106.5	54.5	Yes
	1.273101	138	92	No
progestin-only intrauterine device	0.958248	113	78.5	Yes
	1.253936	111	57.5	No
	1.253936	121	67.5	No
progestin-only intrauterine device	1.253936	107	67	No
progestin-only intrauterine device	1.245722	110.5	64	No
	1.237509	114.5	74	No
	1.237509	119	67	No
progestin-only intrauterine device	1.237509	123	66.5	No
	1.234771	115.5	70	No
	0.960986	107	65.5	No
	1.234771	131	75.5	No
oral hormonal contraception	1.152635	133.5	81.5	No
	1.232033	113	59.5	No
	1.232033	111	67	No
progestin-only intrauterine device	1.193703	118	69.5	No
oral hormonal contraception	1.193703	115	66	Yes
	1.193703	101.5	70.5	No
	1.190965	119	78	No
	1.190965	129.5	75.5	No
progestin-only intrauterine device	1.190965	136.5	71	No
	1.180014	137.5	76.5	No
	1.073238	124.5	71	Yes
	1.004791	120.5	66.5	No
progestin-only hormonal implants	1.059548	124.5	84.5	No
	1.174538	120	73	Yes
	1.174538	130.5	74.5	No
progestin-only intrauterine device	1.1718	127.5	68	No
	1.059548	102.5	64	No
	1.1718	116	48.5	No
oral hormonal contraception	1.158111	113	68.5	No
	1.158111	133	83.5	Yes
progestin-only intrauterine device	1.155373	122	78	Yes
	1.155373	116	76	No
	1.155373	119.5	74.5	No
	1.152635	113.5	72	No
	1.034908	122	73.5	No
progestin-only intrauterine device	1.138946	112	67	No
	1.138946	128	72	No
	1.138946	113.5	69.5	No
	0.81588	106	64	Yes
	1.054073	119.5	70	No
progestin-only intrauterine device	1.034908	110	64.5	No
	1.075975	113.5	64.5	No
progestin-only hormonal implants	1.13347	136	77.5	Yes
	1.075975	125	70	No
progestin-only intrauterine device	1.073238	116.5	70	Yes
progestin-only intrauterine device	1.05681	119.5	72	No
	1.054073	121.5	83	No

	1.021218	113.5	67	Yes
	1.021218	118.5	78.5	No
	1.021218	138	86	No
injectable progestin-only preparations	1.01848	120.5	75.5	Yes
progestin-only intrauterine device	1.01848	120	83.5	No
progestin-only intrauterine device	1.01848	140	81	No
	1.007529	144	69	No
	0.77755	108.5	60	No
	1.004791	121.5	73.5	No
	0.985626	138.5	87	No
	0.988364	144.5	85.5	No
progestin-only intrauterine device	0.988364	122	67.5	No
	0.988364	137.5	87.5	No
injectable progestin-only preparations	0.985626	116	66	No
	0.985626	133.5	81	No
	0.982888	101.5	59.5	Yes
	1.385352	114.5	68.5	No
oral hormonal contraception	0.982888	122	68	Yes
progestin-only intrauterine device	0.974675	154	98.5	No
oral hormonal contraception	0.758385	112	61.5	Yes
	0.930869	131.5	84	No
	0.930869	108.5	67	Yes
	0.922656	125.5	77	No
oral hormonal contraception	0.922656	113.5	60.5	Yes
	0.826831	137.5	79.5	No
progestin-only intrauterine device	0.911704	118	77	No
oral hormonal contraception	0.911704	103	57	No
	0.908966	114.5	64	No
	0.908966	116.5	75.5	No
	0.908966	128	81.5	Yes
progestin-only intrauterine device	0.906229	123	66.5	No
progestin-only intrauterine device	0.906229	119.5	75.5	Yes
progestin-only intrauterine device	0.906229	110.5	70.5	No
	0.903491	119	76	No
progestin-only intrauterine device	0.892539	111	72	No
	0.892539	129.5	77.5	Yes
	0.892539	152.5	96.5	No
	0.887064	154	97	No
	0.887064	128	75	No
	0.887064	124.5	75.5	No
	0.884326	120	70.5	Yes
progestin-only intrauterine device	0.81588	137	76.5	No
	0.884326	114	58.5	No
	0.835044	113	70	Yes
oral hormonal contraception	0.824093	138.5	74.5	No
	0.835044	107	64	No
	0.832307	121	69.5	No
progestin-only intrauterine device	0.824093	119	65	No
	0.793977	122	72.5	No
	0.766598	108	68.5	No

	0.810404	106.5	63.5	No
	0.810404	153.5	85	Yes
	0.810404	117.5	53	No
	0.791239	123.5	69	No
	0.807666	123	75	No
progestin-only intrauterine device	0.813142	124	70.5	No
	0.807666	125	74	Yes
	0.796715	130	72.5	Yes
	0.758385	134.5	79.5	No
	0.793977	132	64	No
	0.791239	167.5	89	No
	0.788501	125.5	73	No
	0.788501	144	101.5	No
	0.788501	125.5	61	Yes

GT	Date of visit	Date of menstruation
Normal glucose tolerance	19.okt.06	17.okt.06
Normal glucose tolerance	26.okt.06	#NULL!
Normal glucose tolerance	26.okt.06	11.okt.06
Impaired glucose tolerance	01.nov.06	13.okt.06
Normal glucose tolerance	27.okt.06	13.okt.06
Impaired glucose tolerance	27.okt.06	19.okt.06
Normal glucose tolerance	01.nov.06	20.okt.06
Normal glucose tolerance	01.nov.06	27.okt.06
Normal glucose tolerance	02.nov.06	17.okt.06
Impaired glucose tolerance	02.nov.06	31.okt.06
Impaired glucose tolerance	02.nov.06	#NULL!
Normal glucose tolerance	06.nov.06	#NULL!
Impaired glucose tolerance	09.nov.06	28.okt.06
Normal glucose tolerance	09.nov.06	05.nov.06
Normal glucose tolerance	10.nov.06	12.okt.06
Impaired glucose tolerance	10.nov.06	04.nov.06
Impaired glucose tolerance	10.nov.06	30.okt.06
Normal glucose tolerance	07.feb.07	01.feb.07
Impaired glucose tolerance	14.nov.06	02.nov.06
Normal glucose tolerance	15.nov.06	18.okt.06
Normal glucose tolerance	15.nov.06	12.nov.06
Impaired glucose tolerance	22.nov.06	14.nov.06
Normal glucose tolerance	22.nov.06	11.nov.06
Normal glucose tolerance	22.nov.06	29.sep.06
Normal glucose tolerance	24.nov.06	22.nov.06
Normal glucose tolerance	30.nov.06	21.nov.06
Impaired glucose tolerance	06.des.06	23.nov.06
Normal glucose tolerance	06.des.06	01.nov.06
Normal glucose tolerance	07.feb.07	#NULL!
Normal glucose tolerance	24.jan.07	03.jan.07
Normal glucose tolerance	24.jan.07	#NULL!
Normal glucose tolerance	02.okt.07	#NULL!
Normal glucose tolerance	31.jan.07	27.jan.07
Normal glucose tolerance	06.feb.07	19.jan.07
Normal glucose tolerance	06.feb.07	20.jan.07
Impaired glucose tolerance	07.feb.07	#NULL!
Impaired glucose tolerance	07.feb.07	28.jan.07
Normal glucose tolerance	08.feb.07	27.jan.07
Impaired glucose tolerance	08.feb.07	01.feb.07
Normal glucose tolerance	07.feb.07	#NULL!
Impaired glucose tolerance	14.feb.07	04.feb.07
Normal glucose tolerance	14.feb.07	02.feb.07
Normal glucose tolerance	14.feb.07	#NULL!
Normal glucose tolerance	14.feb.07	31.jan.07
Normal glucose tolerance	15.feb.07	#NULL!
Normal glucose tolerance	15.feb.07	05.feb.07
Normal glucose tolerance	15.feb.07	02.feb.07
Normal glucose tolerance	08.mar.07	27.feb.07

Normal glucose tolerance	20.feb.07	16.feb.07
Impaired glucose tolerance	20.feb.07	30.jan.07
Normal glucose tolerance	20.feb.07	18.feb.07
Normal glucose tolerance	05.mar.07	#NULL!
Normal glucose tolerance	05.mar.07	22.feb.07
Impaired glucose tolerance	05.mar.07	17.feb.07
Normal glucose tolerance	06.mar.07	#NULL!
Impaired glucose tolerance	06.mar.07	22.feb.07
Normal glucose tolerance	06.mar.07	#NULL!
Normal glucose tolerance	07.mar.07	#NULL!
Normal glucose tolerance	07.mar.07	05.feb.07
Normal glucose tolerance	07.mar.07	04.mar.07
Normal glucose tolerance	08.mar.07	17.feb.07
Normal glucose tolerance	08.mar.07	03.jan.07
Normal glucose tolerance	14.mar.07	16.feb.07
Normal glucose tolerance	14.mar.07	#NULL!
Normal glucose tolerance	14.mar.07	02.mar.07
Impaired glucose tolerance	20.mar.07	15.mar.07
Impaired glucose tolerance	20.mar.07	18.mar.07
Normal glucose tolerance	21.mar.07	#NULL!
Normal glucose tolerance	21.mar.07	#NULL!
Normal glucose tolerance	22.mar.07	05.mar.07
Diabetes	19.apr.07	19.mar.07
Normal glucose tolerance	26.mar.07	#NULL!
Normal glucose tolerance	26.mar.07	24.mar.07
Normal glucose tolerance	26.mar.07	#NULL!
Impaired glucose tolerance	19.apr.07	06.apr.07
Normal glucose tolerance	29.mar.07	22.mar.07
Normal glucose tolerance	11.apr.07	03.apr.07
Normal glucose tolerance	11.apr.07	#NULL!
Normal glucose tolerance	11.apr.07	20.mar.07
Normal glucose tolerance	24.apr.07	28.mar.07
Impaired glucose tolerance	12.apr.07	#NULL!
Normal glucose tolerance	12.apr.07	08.apr.07
Normal glucose tolerance	12.apr.07	10.apr.07
Normal glucose tolerance	18.apr.07	07.apr.07
Normal glucose tolerance	18.apr.07	11.apr.07
Normal glucose tolerance	25.apr.07	#NULL!
Normal glucose tolerance	19.apr.07	#NULL!
Normal glucose tolerance	19.apr.07	#NULL!
Normal glucose tolerance	22.mai.07	06.mai.07
Normal glucose tolerance	25.apr.07	14.apr.07
Normal glucose tolerance	25.apr.07	#NULL!
Impaired glucose tolerance	25.apr.07	25.feb.07
Normal glucose tolerance	30.apr.07	#NULL!
Impaired glucose tolerance	02.mai.07	24.apr.07
Normal glucose tolerance	02.mai.07	#NULL!
Normal glucose tolerance	02.mai.07	29.apr.04
Impaired glucose tolerance	03.mai.07	#NULL!
Normal glucose tolerance	16.okt.07	18.sep.07

Normal glucose tolerance	22.mai.07	14.mai.07
	06.sep.07	21.aug.07
Normal glucose tolerance	09.mai.07	28.apr.07
Diabetes	09.mai.07	05.mai.07
Normal glucose tolerance	10.mai.07	02.mai.07
Normal glucose tolerance	10.mai.07	#NULL!
Normal glucose tolerance	09.mai.07	04.mai.07
Normal glucose tolerance	15.mai.07	17.sep.05
Normal glucose tolerance	16.mai.07	06.mai.07
Normal glucose tolerance	10.mai.07	26.apr.07
Normal glucose tolerance	24.mai.07	06.mai.07
Normal glucose tolerance	24.mai.07	05.mai.07
Normal glucose tolerance	29.mai.07	14.mai.07
Normal glucose tolerance	29.mai.07	13.mai.07
Normal glucose tolerance	29.mai.07	#NULL!
Normal glucose tolerance	07.jun.07	23.mai.07
Normal glucose tolerance	07.jun.07	24.mai.07
Normal glucose tolerance	12.jun.07	07.jun.07
Diabetes	24.okt.07	#NULL!
Impaired glucose tolerance	20.jun.07	08.jun.07
Normal glucose tolerance	09.jan.08	#NULL!
Normal glucose tolerance	13.jun.07	03.jun.07
Normal glucose tolerance	13.jun.07	#NULL!
Normal glucose tolerance	14.jun.07	11.mai.07
Normal glucose tolerance	18.jun.07	09.jun.07
Impaired glucose tolerance	19.sep.07	#NULL!
Normal glucose tolerance	18.jun.07	17.jun.07
Normal glucose tolerance	27.jun.07	18.jun.07
Normal glucose tolerance	04.sep.07	24.aug.07
Impaired glucose tolerance	27.jun.07	03.jun.07
Normal glucose tolerance	04.sep.07	#NULL!
Normal glucose tolerance	04.sep.07	25.aug.07
Normal glucose tolerance	04.okt.07	03.sep.07
Normal glucose tolerance	18.sep.07	07.sep.07
Normal glucose tolerance	18.sep.07	28.aug.07
Normal glucose tolerance	20.sep.07	17.sep.07
Normal glucose tolerance	26.sep.07	24.aug.07
Normal glucose tolerance	26.sep.07	22.sep.07
Normal glucose tolerance	27.sep.07	23.sep.07
Normal glucose tolerance	27.sep.07	14.sep.07
Normal glucose tolerance	02.okt.07	26.sep.07
Normal glucose tolerance	04.okt.07	17.sep.07
Normal glucose tolerance	04.okt.07	27.sep.07
Normal glucose tolerance	02.okt.07	26.sep.07
Normal glucose tolerance	16.okt.07	28.sep.07
Normal glucose tolerance	16.okt.07	06.okt.07
Normal glucose tolerance	17.okt.07	#NULL!
Normal glucose tolerance	17.okt.07	18.aug.07
Normal glucose tolerance	17.okt.07	#NULL!
Normal glucose tolerance	18.okt.07	03.okt.07

Normal glucose tolerance	18.okt.07	03.okt.07
Diabetes	23.okt.07	15.okt.07
Normal glucose tolerance	15.feb.08	#NULL!
Impaired glucose tolerance	30.okt.07	08.okt.07
Normal glucose tolerance	30.okt.07	16.okt.07
Normal glucose tolerance	30.okt.07	06.okt.07
Normal glucose tolerance	02.nov.07	#NULL!
Normal glucose tolerance	05.nov.07	29.okt.07
Normal glucose tolerance	05.nov.07	23.okt.07
Normal glucose tolerance	05.nov.07	#NULL!
Normal glucose tolerance	06.nov.07	22.okt.07
Normal glucose tolerance	14.feb.08	24.jan.08
Normal glucose tolerance	06.nov.07	29.okt.07
Normal glucose tolerance	06.des.07	25.nov.07
Normal glucose tolerance	07.nov.07	28.okt.07
Normal glucose tolerance	07.nov.07	19.okt.07
Normal glucose tolerance	21.nov.07	08.nov.07
Normal glucose tolerance	21.nov.07	23.okt.07
Normal glucose tolerance	21.nov.07	05.nov.07
Normal glucose tolerance	22.nov.07	13.nov.07
Normal glucose tolerance	22.nov.07	10.nov.07
Normal glucose tolerance	22.nov.07	12.nov.07
Impaired glucose tolerance	26.nov.07	05.nov.07
Normal glucose tolerance	04.jan.08	14.des.07
Normal glucose tolerance	29.jan.08	27.jan.08
Normal glucose tolerance	09.jan.08	#NULL!
Normal glucose tolerance	28.nov.07	17.nov.07
Diabetes	28.nov.07	14.nov.07
Impaired glucose tolerance	29.nov.07	10.nov.07
Normal glucose tolerance	09.jan.08	04.jan.08
Normal glucose tolerance	29.nov.07	15.nov.07
Normal glucose tolerance	04.des.07	#NULL!
Normal glucose tolerance	04.des.07	20.nov.07
Impaired glucose tolerance	05.des.07	#NULL!
Normal glucose tolerance	05.des.07	29.nov.07
Impaired glucose tolerance	05.des.07	17.nov.07
Impaired glucose tolerance	06.des.07	20.nov.07
Normal glucose tolerance	18.jan.08	08.jan.08
Normal glucose tolerance	11.des.07	06.des.07
Normal glucose tolerance	11.des.07	29.nov.07
Normal glucose tolerance	11.des.07	23.nov.07
Normal glucose tolerance	07.apr.08	19.mar.08
Normal glucose tolerance	11.jan.08	31.des.07
Normal glucose tolerance	18.jan.08	15.jan.08
Normal glucose tolerance	03.jan.08	11.des.07
Normal glucose tolerance	13.des.07	#NULL!
Normal glucose tolerance	03.jan.08	27.des.07
Impaired glucose tolerance	04.jan.08	#NULL!
Normal glucose tolerance	10.jan.08	#NULL!
Impaired glucose tolerance	11.jan.08	23.des.07

Normal glucose tolerance	23.jan.08	10.jan.08
Normal glucose tolerance	23.jan.08	09.des.07
Normal glucose tolerance	23.jan.08	07.jan.08
Normal glucose tolerance	24.jan.08	#NULL!
Diabetes	24.jan.08	#NULL!
Normal glucose tolerance	24.jan.08	#NULL!
Impaired glucose tolerance	28.jan.08	02.jan.08
Normal glucose tolerance	21.apr.08	02.apr.08
Impaired glucose tolerance	29.jan.08	21.jan.08
Normal glucose tolerance	05.feb.08	09.jan.08
Normal glucose tolerance	04.feb.08	31.jan.08
Normal glucose tolerance	04.feb.08	#NULL!
Impaired glucose tolerance	04.feb.08	15.des.07
Impaired glucose tolerance	05.feb.08	28.nov.07
Normal glucose tolerance	05.feb.08	#NULL!
Normal glucose tolerance	06.feb.08	20.jan.08
Normal glucose tolerance	12.sep.07	04.jul.07
Normal glucose tolerance	06.feb.08	18.jan.08
Impaired glucose tolerance	09.feb.08	#NULL!
Normal glucose tolerance	28.apr.08	20.apr.08
Normal glucose tolerance	25.feb.08	20.feb.08
Normal glucose tolerance	25.feb.08	12.feb.08
Normal glucose tolerance	28.feb.08	17.feb.08
Normal glucose tolerance	28.feb.08	05.nov.07
Normal glucose tolerance	03.apr.08	09.mar.08
Normal glucose tolerance	03.mar.08	#NULL!
Normal glucose tolerance	03.mar.08	16.feb.08
Normal glucose tolerance	04.mar.08	03.mar.08
Normal glucose tolerance	04.mar.08	09.feb.08
Normal glucose tolerance	04.mar.08	20.feb.08
Normal glucose tolerance	05.mar.08	#NULL!
Normal glucose tolerance	05.mar.08	#NULL!
Normal glucose tolerance	05.mar.08	#NULL!
Impaired glucose tolerance	06.mar.08	24.feb.08
Normal glucose tolerance	10.mar.08	28.feb.08
Normal glucose tolerance	10.mar.08	08.mar.08
Normal glucose tolerance	10.mar.08	15.feb.08
Normal glucose tolerance	12.mar.08	01.mar.08
Impaired glucose tolerance	12.mar.08	22.feb.08
Normal glucose tolerance	12.mar.08	#NULL!
Normal glucose tolerance	13.mar.08	02.mar.08
Impaired glucose tolerance	07.apr.08	#NULL!
Impaired glucose tolerance	13.mar.08	#NULL!
Normal glucose tolerance	31.mar.08	18.mar.08
Normal glucose tolerance	04.apr.08	19.mar.08
Normal glucose tolerance	31.mar.08	12.mar.08
Normal glucose tolerance	01.apr.08	#NULL!
Impaired glucose tolerance	04.apr.08	#NULL!
Impaired glucose tolerance	15.apr.08	07.apr.08
Normal glucose tolerance	25.apr.08	16.apr.08

Normal glucose tolerance	09.apr.08	15.mar.08
Normal glucose tolerance	09.apr.08	10.feb.08
Normal glucose tolerance	09.apr.08	26.mar.08
Diabetes	16.apr.08	25.mar.08
Normal glucose tolerance	10.apr.08	07.apr.08
Normal glucose tolerance	08.apr.08	#NULL!
Normal glucose tolerance	10.apr.08	02.apr.08
Diabetes	14.apr.08	#NULL!
Normal glucose tolerance	28.apr.08	20.apr.08
Normal glucose tolerance	15.apr.08	03.mar.08
Normal glucose tolerance	16.apr.08	10.apr.08
Impaired glucose tolerance	17.apr.08	14.mar.08
Impaired glucose tolerance	17.apr.08	12.apr.08
Normal glucose tolerance	17.apr.08	11.apr.08
