# Supplementary figures and images for: Does AMH Reflect Follicle Number Similarly in Women with and without PCOS?
Source: PLoS One. 2016 Jan 22;11(1):e0146739. doi: 10.1371/journal.pone.0146739 (PMC4723054; doi:10.1371/journal.pone.0146739)

**S1 Fig** Inclusion and exclusion decision tree for the current study


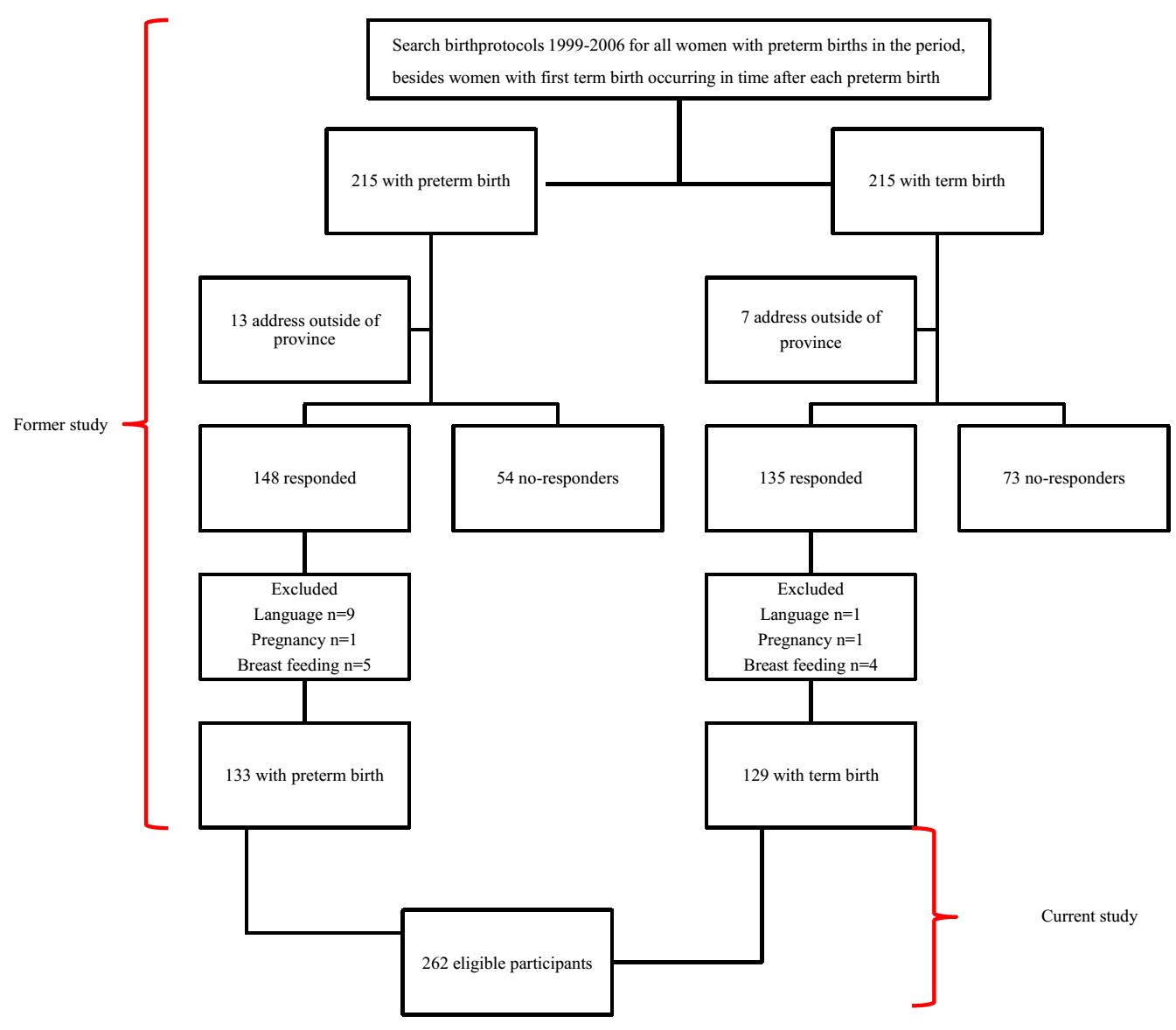

Supplement: S1 Fig — (DOCX) [file pone.0146739.s002.docx]
