# Supplementary material for: Does AMH Reflect Follicle Number Similarly in Women with and without PCOS?
Source: PLoS One. 2016 Jan 22;11(1):e0146739. doi: 10.1371/journal.pone.0146739 (PMC4723054; doi:10.1371/journal.pone.0146739)
Supplement: S2 Fig — Black columns: Women with PCOS. Grey columns: Women with PCOM. White columns: Control women. (DOCX) [file pone.0146739.s003.docx]

**S2 Fig**, Period from blood draw to analysis, in quartiles of the passed period


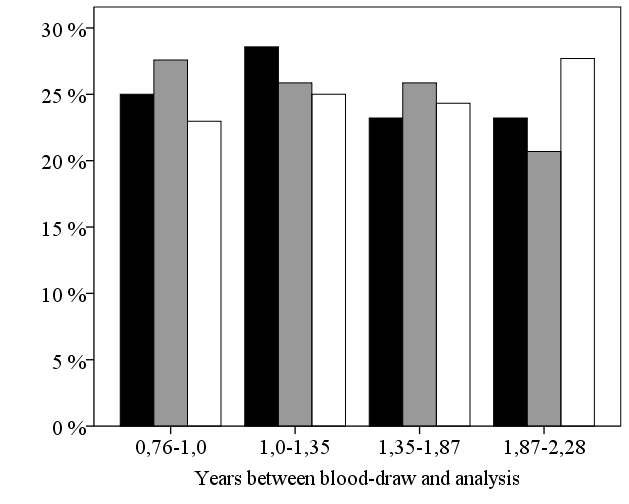


Black columns: Women with PCOS

Grey columns: Women with PCOM

White columns: Control women
