# Supplementary material for: Does AMH Reflect Follicle Number Similarly in Women with and without PCOS?
Source: PLoS One. 2016 Jan 22;11(1):e0146739. doi: 10.1371/journal.pone.0146739 (PMC4723054; doi:10.1371/journal.pone.0146739)
Supplement: S1 Table — Difference compared to controls; Mann Whitney U test for independent samples. (DOCX) [file pone.0146739.s004.docx]

**S1**, Young group 21-35 years (N=140)

|  | PCOS  Mean (SD)  N=33 | P-value* | PCOM  Mean (SD)  N=39 | P-value* | Controls  Mean (SD)  N=68 |
| --- | --- | --- | --- | --- | --- |
|  |  |  |  |  |  |
| AMH (pmol/L)  21-35 years | 50.7 (23.6) | <0.01 | 36.1 (22.2) | <0.01 | 17.4 (12.0) |
| AFC (no.)  21-35 years | 39.7 (14.8) | <0.01 | 28.9 (7.2) | <0.01 | 13.6 (5.1) |
| AMH/AFC ratio  21-35 years | 1.3 (0.4) | 0.29 | 1.3 (0.7) | 0.79 | 1.5 (1.7) |

*Difference compared to controls; Mann Whitney U test for independent samples
