# Supplementary material for: Does AMH Reflect Follicle Number Similarly in Women with and without PCOS?
Source: PLoS One. 2016 Jan 22;11(1):e0146739. doi: 10.1371/journal.pone.0146739 (PMC4723054; doi:10.1371/journal.pone.0146739)
Supplement: S2 Table — Difference compared to controls; Mann Whitney U test for independent samples. (DOCX) [file pone.0146739.s005.docx]

**S2**, Elder group 36-46 years (N=122)

|  | PCOS  Mean (SD)  N=23 | P-value* | PCOM  Mean (SD)  N=19 | P-value* | Controls  Mean (SD)  N=80 |
| --- | --- | --- | --- | --- | --- |
|  |  |  |  |  |  |
| AMH (pmol/L)  36-46 years | 36.4 (31.0) | <0.01 | 26.0 (14.5) | <0.01 | 12.1 (11.0) |
| AFC (no.)  36-46 years | 30.2 (13.7) | <0.01 | 24.6 (5.6) | <0.01 | 11.6 (4.6) |
| AMH/AFC ratio  36-46 years | 1.1 (0.6) | 0.34 | 1.0 (0.4) | 0.58 | 1.1 (1.1) |

*Difference compared to controls; Mann Whitney U test for independent samples
