# Supplementary material for: Does AMH Reflect Follicle Number Similarly in Women with and without PCOS?
Source: PLoS One. 2016 Jan 22;11(1):e0146739. doi: 10.1371/journal.pone.0146739 (PMC4723054; doi:10.1371/journal.pone.0146739)
Supplement: S3 Table — Mann Whitney U test for independent samples. (DOCX) [file pone.0146739.s006.docx]

**S3**, Values from table S1 and S2: Young group versus elder group

|  | PCOS  N= 33 vs N=23 | PCOM  N= 39 vs N=19 | Controls  N= 68 vs N=80 | |
| --- | --- | --- | --- | --- |
|  |  |  |  |  |
| AMH (pmol/L) | <0.01 | 0.08 | <0.01 |  |
| AFC (no.) | <0.01 | 0.02 | 0.01 |  |
| AMH/AFC ratio | 0.21 | 0.23 | 0.04 |  |

* Mann Whitney U test for independent samples
