# Supplementary material for: Does AMH Reflect Follicle Number Similarly in Women with and without PCOS?
Source: PLoS One. 2016 Jan 22;11(1):e0146739. doi: 10.1371/journal.pone.0146739 (PMC4723054; doi:10.1371/journal.pone.0146739)
Supplement: S4 Table — (DOCX) [file pone.0146739.s007.docx]

**S4**, Method of contraception in 90 women who used contraception with hormones

|  | PCOS  N (% of group) | PCOM  N (% of group) | Controls  N (% of group) |  |
| --- | --- | --- | --- | --- |
| oral hormonal contraception | 1 (1.8) | 7 (12.1) | 17 (11.5) | 25 |
| injectable progestin-only preparations | 2 (3.6) | 3 (5.2) | 6 (4.1) | 11 |
| progestin-only hormonal implants | 1 (1,8) | 1 (1.7) | 1 (0.7) | 3 |
| dermal contraception with progestin and estrogen | 0 | 0 | 1 (0.7) | 1 |
| progestin-only intrauterine device | 8 (14.3) | 10 (17.2) | 32 (21.6) | 50 |
|  | 12 (21.4) | 21 (36.2) | 57 (38.5) | 90 |
