# Supplementary material for: Does AMH Reflect Follicle Number Similarly in Women with and without PCOS?
Source: PLoS One. 2016 Jan 22;11(1):e0146739. doi: 10.1371/journal.pone.0146739 (PMC4723054; doi:10.1371/journal.pone.0146739)
Supplement: S5 Table — Difference compared to controls; Mann Whitney U test for independent samples. (DOCX) [file pone.0146739.s008.docx]

**S5**, Mean systolic and diastolic blood pressure, mean of last 2 of 3 measurements

|  | PCOS  Mean (SD) | p | PCOM  Mean (SD) | p | Controls  Mean (SD) |
| --- | --- | --- | --- | --- | --- |
| Systolic BP | 122.3 (13.3) | 0.95 | 118.9 (11.4) | 0.09 | 122.0 (12.4) |
| Diastolic BP | 72.9 (11.1) | 0.88 | 72.2 (9.5) | 0.45 | 72.9 (9.3) |

*Difference compared to controls; Mann Whitney U test for independent samples
