# Supplementary material for: Does AMH Reflect Follicle Number Similarly in Women with and without PCOS?
Source: PLoS One. 2016 Jan 22;11(1):e0146739. doi: 10.1371/journal.pone.0146739 (PMC4723054; doi:10.1371/journal.pone.0146739)
Supplement: S6 Table — (DOCX) [file pone.0146739.s009.docx]

**S6**, The prevalence of diabetes, impaired glucose tolerance (IGT), and normal glucose tolerance (NGT) among women with PCOS, PCOM, and in controls

|  | Diabetes | IGT | NGT | Sysmis |  |
| --- | --- | --- | --- | --- | --- |
| 56 PCOS-women | 1 (1.8%) | 12 (21.4 %) | 43 (76.8 %) | 0 | 56 |
| 58 PCOM-women | 5 (8.6 %) | 9 (15.5 %) | 43 (74.1 %) | 1 | 58 |
| 148 Controls | 2 (1.4 %) | 27 (18.2 %) | 119 (80.4 %) | 0 | 148 |
|  | 8 | 48 | 205 | 1 | 262 |
