# Supplementary material for: Does AMH Reflect Follicle Number Similarly in Women with and without PCOS?
Source: PLoS One. 2016 Jan 22;11(1):e0146739. doi: 10.1371/journal.pone.0146739 (PMC4723054; doi:10.1371/journal.pone.0146739)
Supplement: S7 Table — Difference compared to controls; Mann Whitney U test for independent samples. Pearson’s chi-square test. (DOCX) [file pone.0146739.s010.docx]

**S7** Table, mean age, AMH, AFC, AMH/AFC-ratio, and prevalence PCOS among those who delivered preterm and at term.

|  | Preterm birth  Mean (SD)  N=133 | Term birth  Mean (SD)  N=129 | P-value* |
| --- | --- | --- | --- |
| Age | 34.8 (4.9) | 34.9 (5.4) | 0.59 |
| AMH (pmol/L) | 25.9 (23.6) | 24.2 (20.5) | 0.72 |
| AFC (no.) | 22.2 (14.2) | 19.3 (11.6) | 0.15 |
| AMH/AFC ratio | 1.2 (0.8) | 1.3 (1.4) | 0.45 |

*Difference compared to controls; Mann Whitney U test for independent samples

|  | Preterm birth  N=133 | Term birth  Mean (SD)  N=129 | P-value* |
| --- | --- | --- | --- |
| Prevalence PCOS | 27.8 % | 14.7 % | <0.01** |

**Pearson’s chi-square test
